# Assessing BME688 Sensor Performance Under Controlled Outdoor-like Environmental Conditions

**DOI:** 10.3390/s25237102

**Published:** 2025-11-21

**Authors:** Enza Panzardi, Ada Fort, Valerio Vignoli, Irene Cappelli, Luigi Gaioni, Matteo Verzeroli, Salvatore Dello Iacono, Alessandra Flammini

**Affiliations:** 1 Department of Information Engineering and Mathematical Science, University of Siena, 53100 Siena, Italy; ada.fort@unisi.it (A.F.); valerio.vignoli@unisi.it (V.V.); irene.cappelli2@unisi.it (I.C.); 2 Department of Engineering and Applied Sciences, University of Bergamo, 24129 Bergamo, Italy; luigi.gaioni@unibg.it (L.G.); matteo.verzeroli@unibg.it (M.V.); 3 Department of Information Engineering, University of Brescia, 25121 Brescia, Italy; salvatore.delloiacono@unibs.it (S.D.I.); alessandra.flammini@unibs.it (A.F.)

**Keywords:** gas sensors, MOX gas sensors, gas sensor characterization, air quality, pollution monitoring

## Abstract

**Highlights:**

**What are the main findings?**

- BME688 sensors show consistent normalized responses across devices but require individual baseline calibration.
- Each gas (NO_2_, CO, SO_2_, isobutylene) has an optimum operating temperature in the 360–400 °C range, with fast and stable responses.
- Humidity enhances CO sensing via hydroxyl pathways but reduces sensitivity and stability for other gases.

**What is the implication of the main finding?**

- BME688 can be deployed in outdoor/mobile air quality networks if calibration and compensation for temperature/humidity are applied.
- The findings support robust, low-cost IoT platforms bridging lab characterization and real-world monitoring.

**Abstract:**

Low-cost miniaturized gas sensors are increasingly considered for outdoor air quality monitoring, yet their performance under real-world environmental conditions remains insufficiently characterized. This work evaluates the dynamic gas response of the Bosch BME688 sensor, whose metal oxide sensing layer is based on tin dioxide (SnO_2_) material, focusing on its sensitivity, selectivity, and dynamic response to four representative air pollutants: nitrogen dioxide (NO_2_), carbon monoxide (CO), sulfur dioxide (SO_2_), and isobutylene. This study provides both quantitative performance metrics and a physicochemical interpretation of the sensing mechanism. Controlled experiments were conducted in a custom test chamber to facilitate the precise regulation of temperature, humidity, and gas concentrations in the ppm to sub-ppm range. Despite large variability in the baseline resistance across devices, normalization yields consistent behavior, enabling cross-sensor comparability. The results show that the optimum operating temperatures fall in the range of 360–400 °C, where response and recovery times are reduced to a few minutes, compatible with mobile sensing requirements. Moreover, humidity strongly influences sensor behavior: it generally decreases sensitivity but improves kinetics, and in the case of CO, it enables enhanced responses through additional hydroxyl-mediated pathways. These findings confirm the feasibility of deploying BME688 sensors in distributed outdoor monitoring platforms, provided that humidity and temperature effects are properly addressed through calibration or compensation strategies. In addition, the variability observed in baseline resistance highlights the need for normalization and, consequently, individual calibration steps for each sensor under reference conditions in order to ensure cross-sensor comparability. The findings provided in this study provide support for the design of robust, low-cost air monitoring networks.

## 1. Introduction

In recent years, there has been growing interest in the development of low-cost, miniaturized gas sensors for environmental monitoring, both in academic research and industrial innovation [1]. The ability to deploy distributed sensor networks across urban and rural areas would open up new opportunities for real-time high-resolution air quality mapping. A particularly appealing scenario involves the integration of such sensors into transportation systems, including bicycles, cars, and public vehicles, enabling mobile, scalable, and flexible air pollution monitoring at city scale [2].

If compact, low-cost, and low-power gas sensors were available with sufficient sensitivity and robustness, it would be possible to massively scale environmental data collection, democratizing access to air quality information. This vision has driven a substantial body of research and industrial development aiming to create gas sensing solutions that are suitable for deployment in large numbers, including in wearables or on mobile sensing platforms [3,4].

To date, the only viable approach for sensor miniaturization has been the use of conductivity-based devices, which detect gases through variations in electrical conductivity caused by surface interactions with analytes. These sensors typically consist of films of semiconducting metal oxides (e.g., SnO_2_, WO_3_, ZnO) deposited on micromachined substrates equipped with integrated microheaters. Operating temperatures typically range from 300 °C to 450 °C, the range necessary to activate surface reactions and maintain sensor responsiveness. As a result, major industrial players have invested in this technology and released commercial sensors, primarily targeting indoor air quality applications (e.g., volatile organic compound (VOC) detection, CO_2_ proxies, breath sensing).

More recently, however, the research community has begun exploring the use of these miniaturized MOX sensors in outdoor environments, leveraging their size, affordability, and sensitivity for broader environmental monitoring [5]. One such sensor is the BME688 [6], developed by Bosch (Bosch Sensortec GmbH, Reutlingen, Germany) which combines MOX gas sensing with environmental sensing in a highly integrated format.

The BME688 is a metal oxide (MOX) gas sensor integrating gas detection with pressure, humidity, and temperature sensing in a compact package. According to both official Bosch Sensortec documentation [6] and the preliminary analysis by Klibanov and Boldt in [7], the humidity, pressure, and temperature sensors are monolithically integrated on the same MEMS die, while the gas sensing element is implemented on a separate die. The humidity sensing principle appears to be capacitive, based on two capacitor plates and a polymer dielectric layer, although Bosch has not publicly disclosed the detailed materials or fabrication process. At its core, the BME688 relies on a thin film of large-grained tin dioxide (SnO_2_) deposited over a heated micromachined membrane, where electrical conduction is governed predominantly by the surface charge distribution across the grain boundaries. This distribution creates potential barriers at the grain interfaces, which modulate carrier transport and, ultimately, the sensor’s electrical resistance.

The sensor’s microstructure, defined by the manufacturer’s proprietary technology, directly influences its electrical and gas-response characteristics.

The conductivity of SnO_2_ in this granular form is strongly temperature-dependent, both due to the intrinsic energy distribution of charge carriers and the kinetics of surface reactions that govern chemisorption and desorption processes. Any species that adsorbs on the surface and modifies the surface charge density—through chemisorption (e.g., NO_2_, CO, VOCs)—can induce a measurable electrical response.

However, even physisorbed species, present at lower temperatures, may influence the signal, e.g., by screening surface charges, thereby modulating the barrier heights and altering conduction pathways.

Among the compounds that elicit strong sensor responses, water vapor plays a dominant role. Moisture affects both the electronic properties of the surface and the reactivity of other gas species, making ambient humidity a critical factor in outdoor operation. This sensitivity poses a major challenge for deploying MOX sensors like the BME688 in uncontrolled environments such as outdoor deployments, where temperature and humidity vary significantly.

The resistance of the BME688 is, therefore, shaped by two major temperature-related factors: a direct dependence on the thermal excitation of charge carriers, and an indirect dependence via the temperature sensitivity of surface reactions and their kinetics, including adsorption, desorption, and redox processes.

For this reason, it is crucial to understand how and whether the sensor responds to key air pollutants under real-world conditions, particularly if it is to be used for ambient air quality monitoring. Additionally, characterizing the chemical dynamics—namely, the response and recovery times—is essential, because even though the sensor is built on a micromachined membrane with extremely fast thermal time constants, the chemical processes themselves can exhibit intrinsically slower dynamics. This is especially relevant in mobile sensing platforms, where transient exposures and rapid environmental changes require a quick and reliable sensor response.

In this work, we present a detailed investigation of the BME688 operating under outdoor conditions. We aim to evaluate its sensitivity, selectivity, and dynamic response to key atmospheric components in order to assess its suitability for deployment in open-air or mobile environmental monitoring systems. Unlike previous studies, this work provides a physically grounded interpretation of the observed behavior, revealing the true kinetics of the gas–surface interaction. The adopted methodology combines experimental control with physicochemical interpretation to offer a deeper understanding of a device that is attracting major interest in IoT applications. To our knowledge, such a detailed examination of transient processes has not been reported in the scientific literature for this specific sensor.

In detail, we present an experimental characterization of the BME688 sensor by evaluating its sensitivity and dynamic response—including response and recovery times—to a set of representative air pollutants: nitrogen dioxide (NO_2_), carbon monoxide (CO), sulfur dioxide (SO_2_), and isobutylene (as a model for the VOC family). These compounds were selected because they are among the most common and health-relevant components of urban air pollution and because they pose distinct chemical challenges to MOX-based detection.

We also investigate the interactions between gas response and ambient humidity, with particular attention to how water vapor modulates sensitivity and signal stability. The relative humidity was maintained at 50% RH to emulate typical ambient conditions and ensure measurement reproducibility. This preliminary analysis aimed to isolate the individual gas responses; therefore, interactions between gases (e.g., CO–NO_2_–H_2_O) were not explored in this stage and will be systematically investigated in future works.

Cross-sensitivity is an intrinsic feature of metal oxide gas sensors, and testing individual representative gases is the standard way to evaluate it. The selected analytes (CO, NO_2_, SO_2_, isobutylene) reflect the main urban pollutants, and their individual characterization reveals how strongly they interfere with one another. This study also highlights that the BME688 is sufficiently sensitive to detect pollutant concentrations near their health-related limits, a non-trivial result given the small toxic thresholds of some species and the limited resolution of other low-cost technologies such as commercial electrochemical sensors.

The concentration ranges for each gas were carefully chosen to be representative of real-world exposure levels, in line with the typical ambient concentrations encountered in urban environments. In addition to this metrological characterization, we also aim to provide a chemical–physical interpretation of the observed responses. By analyzing how surface interactions and temperature-dependent kinetics influence the sensor signals, we seek to offer a more generalizable understanding of the mechanisms at play. This interpretive framework is essential for improving both sensor calibration and deployment strategies in distributed, low-cost mobile platforms for environmental monitoring, enabling more robust and context-aware interpretations of sensor signals in the field.

Although most research aims to reduce the operating temperature of MOX sensors, in this study, the BME688 was intentionally driven over the full temperature range (200–400 °C) to characterize its intrinsic surface kinetics; however, only the results corresponding to the usable range have been reported. Although the authors have previously explored low-temperature operation strategies [8,9], here, the focus is on high-temperature dynamic behavior to provide the physical basis for the subsequent optimization of low-power sensing.

## 2. Characterization of a Low-Cost MOX Sensor in a Controlled Environment

### 2.1. Measurement Setup and Gas Characterization System

**Figure 1:**
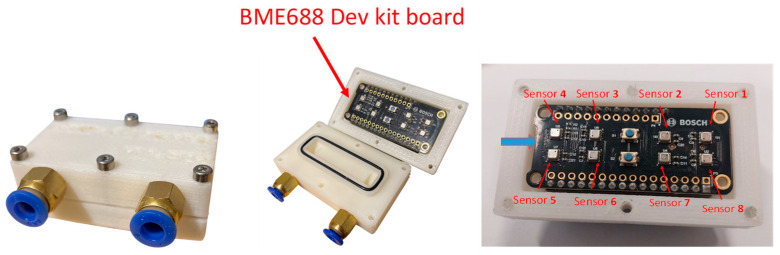
Gas sensor measurement chamber shown in both closed and open configurations, with the BME688 Development Kit housed inside, which embeds the 8 identical BME688 gas sensors.

To experimentally evaluate the performance of the BME688 gas sensor in a controlled measurement environment, a BME688 Development Kit from Bosch Sensortec was employed [10], and the board can be observed in Figure 1. This evaluation platform integrates eight identical BME688 sensors on a single board, each featuring the same metal oxide sensing element, microheater, and integrated circuitry. The use of multiple sensors does not rely on different materials or custom designs; rather, it enables parallel measurements under identical environmental conditions. Indeed, the development kit architecture is designed to support AI-based air quality analysis through Bosch’s BME AI-Studio (v1.3) software and BSEC library [6,10]. The inclusion of multiple identical sensors facilitates the collection of richer datasets, which can be used to train and validate application-specific machine learning models for gas or air-quality recognition.

The development kit offers substantial flexibility in acquiring the raw chemical-resistance data necessary to accurately characterize the gas-sensing performance of each sensor sample in different measurement conditions. Users can define the sampling interval and adjust the sensor operating temperature at each sampling point. This functionality enables the implementation of dynamic temperature profiles; however, it is limited to a fixed sequence of up to ten temperature values per measurement cycle. While the system supports a broad range of gas-sensing scenarios, its constraints on temperature-profile length and sampling frequency should be considered when designing experiments.

For the purposes of this study, the sensor temperature was kept constant during each measurement cycle, with values in the admissible range as desired for different tests. To facilitate the observation of the gas response and minimize the influence of fluid dynamics, a custom measurement chamber was specifically designed for the application [11]. The test chamber was custom-designed and fabricated using 3D-printed ABS, and it is shown in Figure 1. This chamber was engineered to enclose the Device Under Test (DUT), effectively minimizing leakage and ensuring a more direct flow of gas to the sensing area. By reducing the internal dead volume, the system allows for fast gas exchanges, enabling the evaluation of the chemical response/recovery time of the sensors to the different target gases.

The whole measurement process is remotely managed by a Virtual Instrument (VI) developed in the LabVIEW environment. This interface enables the configuration of the BME688 sensors’ operating conditions, such as the working temperature and sampling time, set as 140 ms in this work, and allows for the continuous acquisition as well as the visualization and saving of raw resistance data and other working parameters such as humidity, pressure, and heater current. Communication with the DUT is established via USB (virtual RS232).

In addition, the VI also controls the gas system that regulates the composition (i.e., the desired concentration of each target gas) and flow of the gas mixtures used for the tests. This includes the remote control of a mass flow controller system (Bronkhorst F-201C), which allows for the mixing of accurately regulated gas flows—up to four test gases—to obtain mixtures with known compositions and total flow. During all measurements reported in this paper, the total gas flow rate was maintained at 200 mL/min. The desired gas mixtures at known concentrations were generated by mixing different flow rates originated from two certified reference cylinders: one containing the target gas (e.g., NO_2_) at a certified concentration, and the other containing ultrapure dry air, used as the carrier gas. The concentration of all cylinders was guaranteed by the gas supplier to be within ±2% of the nominal value.

As an example, for the NO_2_ measurements, we used a certified cylinder containing 2 ppm NO_2_ in air and another cylinder of ultrapure air. By mixing 100 mL/min from the NO_2_ cylinder with 100 mL/min from the carrier gas cylinder, we obtained a final mixture with a NO_2_ concentration of 1 ppm while maintaining the total flow at 200 mL/min by exploiting the accurate dilution of the original cylinder. This approach enables the production of a wide range of concentrations from a limited set of certified reference cylinders, ensuring both flexibility and cost-efficiency. The accuracy of the mass flow controllers, specified as ±0.5% of the reading plus ±0.1% of the full scale, ensures that the generated concentrations remain within an acceptable uncertainty range. Ultrapure air was used as the carrier gas in order to ensure accurate and reproducible measurements because it provides a clean reference atmosphere, free from interfering species such as CO_2_, VOCs, NO_x_, PMx, or uncontrolled water vapor content, which could otherwise affect the baseline signal and lead to non-reproducible results.

The tests reported in this work evaluate the sensor response to nitrogen dioxide (NO_2_; original cylinder of 2 ppm concentration in air), isobutylene (C_4_H_8_; original cylinder of 2 ppm concentration in air), carbon monoxide (CO; original cylinder of 20 ppm concentration in air), and sulfur dioxide (SO_2_; original cylinder of 40 ppm concentration in air). These compounds were selected as representative outdoor air pollutants. In particular, NO_2_, CO, and sulfur dioxide (SO_2_) are classified by the U.S. Environmental Protection Agency (EPA) as criteria air pollutants, ubiquitous components of ambient air that are stringently regulated due to their documented adverse effects on human health and the environment [12], whereas isobutylene is a gas representative of VOCs commonly emitted in urban and industrial environments, contributing to atmospheric photochemical reactions and serving as a typical tracer of industrial hydrocarbon emissions [13].

The humidity levels inside the test chamber were adjusted using a bubbler system filled with ultrapure water. A dry synthetic air (or nitrogen) stream was passed through the bubbler to achieve saturation, and the relative humidity (RH) levels were modulated by mixing this saturated airflow with the dry mixtures in varying proportions. All tests were performed at 50% RH, a representative and easily controllable condition corresponding to typical ambient humidity levels.

**Figure 2:**
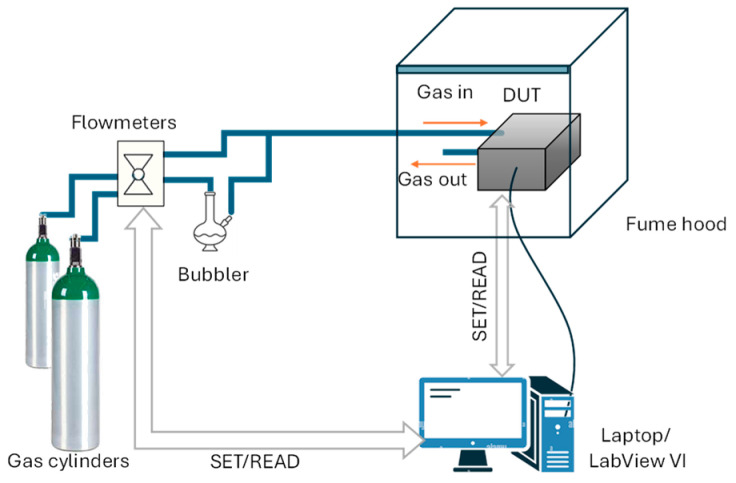
Sensor characterization setup.

In Figure 2, a schematic diagram of the measurement and characterization setup is reported.

The experimental dataset was collected over a measurement campaign lasting several months, during which repeated exposures were performed under controlled conditions of temperature, humidity, and gas composition. It should be underlined that, although the BME688 sensors were characterized over a wide temperature range (200–400 °C), only the responses obtained above approximately 240 °C are reported in the following sections. Indeed, at lower temperatures, the response and recovery kinetics were extremely slow, requiring several tens of minutes to approach steady state. Because such conditions are not compatible with dynamic sensing applications (e.g., mobile or wearable systems), these data were excluded from the investigation of the sensors’ responses to the tested gases. Therefore, the temperature range shown corresponds to the region of practical operation, where the sensors display measurable and reliable gas differentiation.

### 2.2. Experimental Results: Gas Response

Taking into account the structure of the sensing film [7], which consists of a loosely connected micro-grained layer, the sensing mechanism is primarily related to the formation of a potential barrier at the grain surface, which determines the conductivity. The sensing material is SnO_2_, which behaves as an n-type semiconductor.

In some more detail, for this kind of sensing film, the height of the potential barrier, V_S_, can be obtained from a 1D description of the depleted region beneath the grain surface. This region is typically formed due to the adsorption of negatively charged species, such as oxygen ions (e.g., O^−^, O_2_^−^), on the grain surface of the n-type semiconductor. These species extract electrons from the conduction band, creating a space charge region (also called the depletion layer) near the surface.

The potential barrier V_S_ represents the energy barrier that electrons must overcome to move from one grain to another; thus, it determines the electrical conductivity of the film. Under the assumption of large grains and a fully depleted surface, the barrier height depends directly on the surface density of the negatively charged species, hereafter denoted as N_s_, as follows [14]:

(1) $$V_{s}=\frac{qN_{s}^{2}}{2n\epsilon },$$

where n is the bulk donor volume density, ϵ is the electrical permittivity of the bulk material, and q is the electron charge.

The resistance of the film is then derived from the density of the carriers able to overcome the potential barrier through thermal energy, and it can be expressed as

(2) $$R=R_{0}e^{\frac{qV_{s}}{kT}},$$

where *R*_0_ is the baseline resistive value of the film, a parameter that depends on the film geometry and the material bulk properties; *k* is the Boltzmann constant; and *T* is the absolute temperature.

The charged species surface density can be written as follows:

(3) $$N_{s}=N_{i}+\left[Y_{ads}^{-}\right]-[X_{ads}^{+}]$$

where N_i_ indicates the density of negatively ionized intrinsic surface defects (considered to be the only type present) and *X* and *Y* represent species adsorbed from the gas phase onto specific surface sites and subsequently ionized, where the squared brackets [ ⋅ ] indicate the surface density of the given species.

From Equations (1) to (3), the overall behavior of the sensor can be understood: Chemisorbed species either increase the barrier height (oxidizing gases) or decrease it (reducing gases), thereby producing a highly nonlinear effect on the film resistance. This simple model clarifies that the sensor response to a gas depends on the chemisorption reaction, on the baseline value of *N_s_* (ionized intrinsic defects), and on the bulk donor density. Importantly, temperature influences resistance both explicitly (through the exponential factor in (2)) and indirectly (by affecting adsorption equilibria and the ionization of surface species) [14,15].

It should be noted that, although the simplified model of Equations (1)–(3) can be applied to the material of the BME688, neither the magnitude of the response nor its dynamics can be predicted, because the reaction rate constants, the baseline values of Ns and n, and their variations all depend on material defects (both bulk and surface), not solely on the intrinsic properties of the pure material. The defect chemistry ultimately depends on the specific preparation route. Therefore, the actual behavior cannot be predicted a priori and must be determined experimentally.

**Figure 3:**
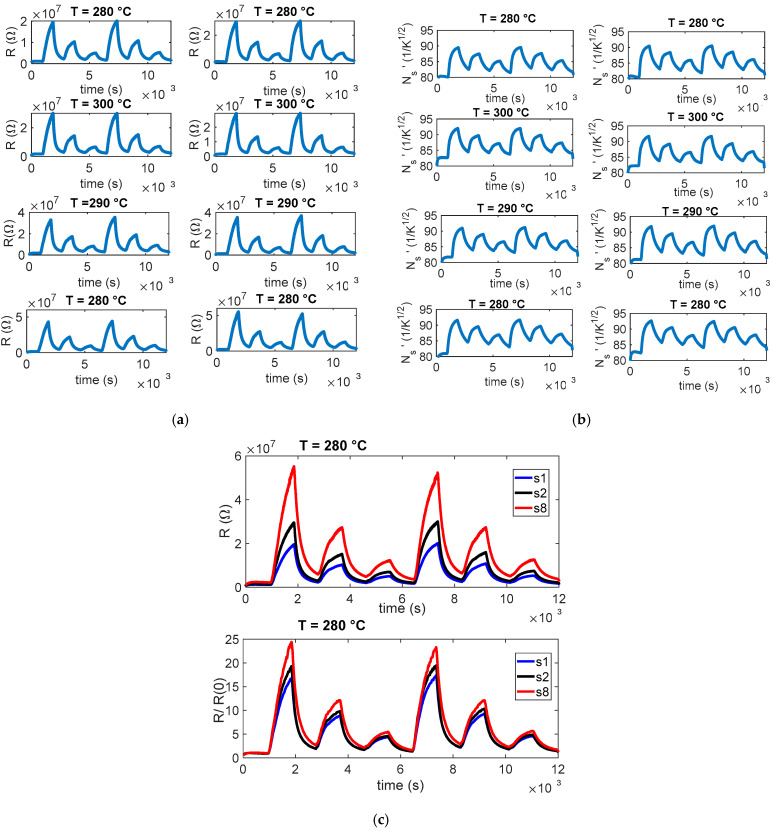
Example of gas measurement results. (**a**) Measured resistance (R) as a function of time for the 8 sensors during a typical measurement session (target gas: NO_2_; concentration pulses of 2 ppm, 1 ppm, and 0.5 ppm followed by pure air recovery phases). The sensing layer temperature is the same for couples of sensors, as shown by the subplot titles. (**b**) Estimated value of N_s_’ = $\frac{\sqrt{q}N_{s}}{\sqrt{n\epsilon }}$. (**c**) Upper plot: measured resistance versus time for sensors 1, 2, and 8 (e.g., 60% average difference s1–s8); lower plot: normalized resistance for the same sensors 1, 2, and 8, (13% average difference s1–s8), where R(0) is the resistance at the beginning of the test (i.e., its baseline reference value in carrier gas).

The planning and execution of the tests must be accurate, as they are often lengthy and time-consuming. In this work, we evaluate the sensor response by exposing it for a fixed duration (15 min) to gas mixtures containing different concentrations of the target gas, diluted in synthetic air under either dry conditions or in the presence of water vapor. Each exposure phase is followed by a recovery phase in clean air, which allows the sensor to approach its baseline resistance. An example of the characterization results obtained following this protocol is shown in Figure 3. Each test was repeated at least twice, and pairs of the eight sensors were always tested under the same operating conditions (same temperature).

The normalized sensor response is quantified using the following parameter:

(4) $$Resp=\frac{R\left(t_{fin}\right)-R(0)}{R(0)}$$

Here, by denoting with R(t) the time-dependent resistance of the sensor, the normalized response parameter *Resp* represents the relative variation in resistance between the beginning and the end of the gas-exposure phase, where $R\left(t_{fin}\right)$ is the resistance at the end of a 15 min exposure phase to the target-gas mixture and *R(0)* is the resistance at the beginning of the test (i.e., its baseline reference value in carrier gas).

This formulation captures the fractional resistance change induced by the target gas. For reducing gases, *Resp* values range between 0 and 1 (decreasing resistance), whereas for oxidizing gases, they are greater than 1 (increasing resistance), in accordance with the expected behavior of SnO_2_-based resistive sensors.

It is worth noting that, especially at low temperatures, the resistance may not reach a steady-state value within the 15 min exposure window. As a result, the calculated response may underestimate the true steady-state response. However, in practice, when the response times at low temperatures are significantly longer than 15 min, the exact steady-state value is of limited relevance. This is because such low temperatures are outside the intended operational range for mobile or real-time applications, where long stabilization times are incompatible with requirements. Therefore, performance at these low temperatures is not considered interesting for practical use.

We emphasize that the same definition of sensor response was maintained for both oxidizing and reducing gases. Naturally, with this definition, the maximum response to a reducing gas is bounded at −1, whereas the response to an oxidizing gas is not upper-limited. For a fairer comparison between the two cases, resistance should ideally be used for oxidizing gases and conductivity for reducing ones. However, this would introduce additional complexity in data presentation, because some gases may switch behavior depending on the operating temperature. In any case, the artifacts introduced by adopting a single unified definition are effectively resolved by examining the response plots, which clearly illustrate the underlying sensor behavior.

As already observed in [11], the eight sensors have very different baseline values (in some conditions, differences larger than 100%), but the normalized resistance shows consistent behaviors (difference within 30%). Such behavior can be seen in the examples reported in Figure 3. Specifically, panel (a) reports the measured resistance values R for the eight sensors; panel (b) shows an estimation of the normalized surface charge density, N_s_’=$\frac{\sqrt{q}N_{s}}{\sqrt{n\epsilon }}$; and panel (c) compares three representative sensors, those with the highest, intermediate, and lowest baseline values, highlighting how normalization with respect to the baseline reduces variability across devices. In our procedure, $N_{s}$ is retrieved in two steps. First, $R_{0}$ is estimated by inverting (2) during rapid temperature steps performed at low temperature, where the density of surface states $N_{s}$ can be treated as constant because the thermal response of the sensing micro-membrane (milliseconds) is much faster than the surface kinetics (minutes). Using T just before and just after this temperature step, we solve for $R_{0}$ from (2). Second, with $R_{0}$ known, $N_{s}(t)$ is obtained over the full trajectory by rearranging (2) and solving for $N_{s}$ from the measured R(t) and the known T(t). This time-scale-separation approach yields a robust and physically consistent estimation of the surface-state density.

As already stated, after normalization, the variability among the eight tested sensors is significantly reduced and confined to levels that are acceptable for detection tasks, even though the absolute accuracy of the concentration measurements remains limited. This indicates that the BME688, when properly normalized, can provide reliable qualitative information on the presence of and relative variation in target gases across a sensor network. However, to ensure comparability among devices, it is essential to establish the baseline resistance of each sensor under standard reference conditions. This step represents a form of individual calibration that cannot be avoided, because baseline values are strongly influenced by intrinsic material defects and manufacturing variability. Defining and standardizing such a calibration procedure by specifying the reference environment, temperature, and humidity conditions is, therefore, a necessary prerequisite for large-scale deployments. By implementing this individual calibration step, the reduced variability achieved through normalization can be effectively exploited, enabling the use of low-cost MOX sensors for distributed monitoring applications. In more detail, before gas exposure, the dynamic resistance trend in synthetic air was analyzed to evaluate the baseline stability and transient effects associated with the heating cycles. The results show gradual stabilization of the baseline resistance over time, mainly attributed to the thermal equilibration of the sensing layer and the desorption of residual surface species. Minor fluctuations observed during steady-state operation reflect the intrinsic dynamic balance between adsorption and desorption processes under ambient conditions. This behavior is consistent with the expected thermal activation of the SnO_2_-based sensing layer and confirms the stable operation of the sensor in air before exposure to target gases.

Because the behavior of the eight sensors in terms of normalized response is acceptably similar and the trend as a function of temperature or of target-gas concentrations is similar and consistent, from now on, the results of a single sensor are presented, but the comments and conclusions drawn can be extended to all the sensors in the batch and, ultimately, to the whole sensor family. In this context, concerning the transient gas test results, the sensor response was expressed as the normalized ratio R/R(0) (R_CHIM_/R(0)) to minimize the effect of baseline differences among the eight sensors. Because each device may exhibit a different initial resistance value, normalization allows researchers to focus on the relative variation induced by gas exposure rather than the absolute resistance, enabling a direct comparison of the dynamic behavior across sensors. Although the normalized responses start approximately from unity, small deviations are observed before gas injection. These deviations arise because the baseline resistance in air is not perfectly stable during the initial phase of each measurement and because normalization with respect to a single reference point can slightly shift the starting level.

#### 2.2.1. NO_2_ Gas Sensor Response

The reaction path usually assumed for NO_2_ sensing is the following [15]:

(5) $$N{O_{2}}_{gas}+e^{-}\Leftrightarrow \;{N{O_{2}}_{ads}}^{-},$$

where $N{O_{2}}_{ads}$ indicates an adsorbed $NO_{2}$ molecule and $e^{-}$ a free electron.

So, NO_2_ behaves as an oxidizing gas and causes an increase in resistance. In fact, considering dry mixtures of air and NO_2_, we can assume for the charge surface density that $N_{s}=N_{i}+\left[Y_{ads}^{-}\right]=N_{i}+\left[O_{ads}^{x-}\right]+\left[N{O_{2}}_{ads}^{-}\right].$ Therefore, the presence of NO_2_ contributes to the increase in $\left[Y_{ads}^{-}\right]$.

Note that $\left[O_{ads}^{x-}\right]$ represents the concentration of chemisorbed oxygen from air.

**Figure 4:**
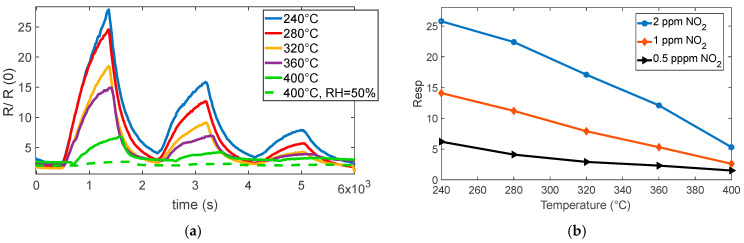
(**a**) Normalized measured resistance as a function of time for sensor 7 during measurement sessions at different operating temperatures (as indicated in the legend) in dry and humid air (NO_2_ gas concentration pulses followed by recovery phases, 2 ppm, 1 ppm, 0.5 ppm). (**b**) Measured response for sensor 7 as a function of the operating temperature for the 3 different concentrations, as indicated in the legend.

The reaction is very favored at the lower temperature, below 360° for instance; at 280 °C, a very large response (as per (4)) of about 8 is found for 0.5 ppm NO_2_. Nevertheless, at this temperature, the reaction kinetics are very slow, as can be seen in Figure 4a.

At temperatures above 360 °C, both the response and recovery times improve significantly, reaching reasonable durations of the order of a few minutes. Although the magnitude of the response decreases (approximately 1 at 0.5 ppm), it remains adequate for detection purposes.

**Figure 5:**
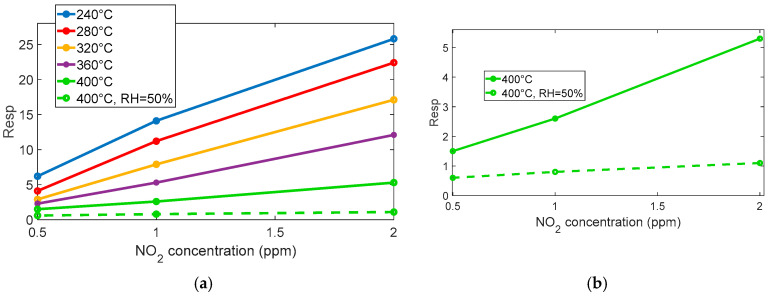
(**a**) Measured response for sensor 7 as a function of NO_2_ concentration for the different temperatures and RH levels, as indicated in the legend. (**b**) Measured response for sensor 7, comparison between response to NO_2_ in dry conditions and in the presence of 50% RH.

**Figure 6:**
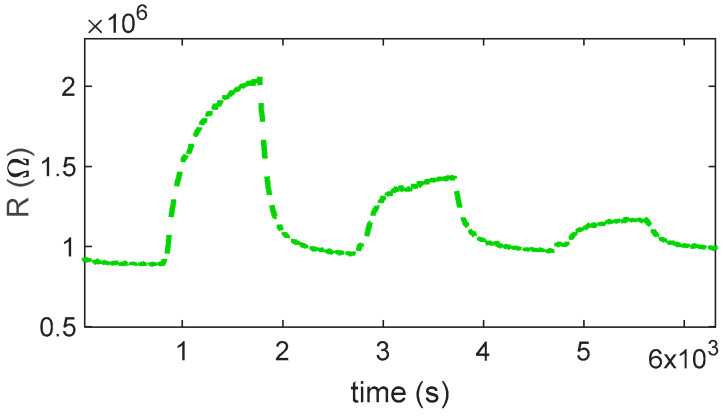
Measured resistance for sensor 7 in the presence of 50% RH as a function of time with target-gas concentration pulses followed by recovery phases, 2 ppm, 1 ppm, and 0.5 ppm at 400 °C.

The presence of water vapor reduces the overall sensor response but simultaneously improves the response speed, as shown in Figure 4a, Figure 5a,b, and Figure 6. Despite the reduced sensitivity, the sensor maintains a detection limit below 0.1 ppm even in the presence of high relative humidity (50%), which confirms its suitability for outdoor air quality monitoring.

In summary, operating the sensor at temperatures above 360 °C and under relative humidity levels exceeding 20% is appropriate and effective for the intended application.

#### 2.2.2. Isobutylene Gas Sensor Response

According to the literature, the sensing mechanism relies on the oxidation of isobutylene by oxygen species that are chemisorbed on the surface of the SnO_2_. This reaction releases electrons back into the conduction band, which, in turn, reduces the height of the grain boundary potential barrier. The reaction route assumed is the following [16]:

(6) $${C_{4}H_{8}}_{gas}+6O_{ads}^{-}\Leftrightarrow 4CO_{2_{gas}}+4H_{2}O_{gas}+{6e}^{-}$$

The preadsorbed oxygen that can intermediate such reaction is that present in the intermediate temperature range [16]:

(7) $${O_{2}}_{gas}+{2e}^{-}\Leftrightarrow \;2O_{ads}^{-}\;\;\;\;\;\;\;\;\;\;\;\;\;(150\;{}^{\circ}C–400\;{}^{\circ}C)$$

As a result, the electrical resistance of the material decreases, with reaction (6) contributing to the decrease in $\left[Y_{ads}^{-}\right]$, as per Equation (3).

At lower temperature, different oxygen species are expected [16]:

(8) $${O_{2}}_{gas}+e^{-}\Leftrightarrow O_{2_{ads}}^{-}\;\;\;(<150\;{}^{\circ}C)$$

**Figure 7:**
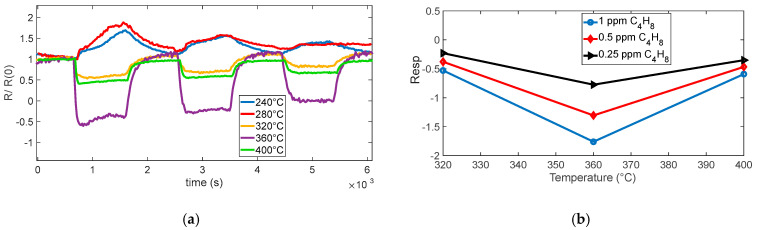
(**a**) Normalized measured resistance as a function of time for sensor 7 during measurement sessions at different operating temperatures (as indicated in the legend) in dry air (target gas concentration pulses followed by recovery phases, 1 ppm, 0.5 ppm, 0.25 ppm). (**b**) Measured response for sensor 7 as a function of the operating temperature for the 3 different concentrations, as indicated in the legend.

**Figure 8:**
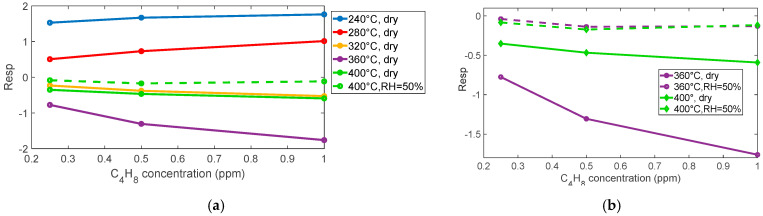
(**a**) Measured response for sensor 7 as a function of isobutylene concentration for the different temperatures and RH, as indicated in the legend. (**b**) Measured response for sensor 7: comparison between response to isobutylene in dry conditions and in the presence of 50% RH.

So, at low temperature, Equation (7) is not the expected reaction. Note that the temperature ranges are approximately known; therefore, for the sensing material used in BME688, the ranges can be different. Specifically, from the measurements (as can be seen in Figure 7 and Figure 8), the expected reducing behavior starts above 300 °C; below, a very slow opposite behavior is registered. Below this temperature, indeed, the ionization kinetics of chemisorbed oxygen species (O_2_^−^, O^−^) and the adsorption–desorption processes of target gases become extremely slow. The surface potential and charge density, therefore, remain nearly constant, resulting in an unstable or negligible resistance variation during gas exposure. Under these conditions, the sensor does not reach steady state within the time of measurement, and the response becomes impractically slow for real-world operation. Above 300 °C, the activation of surface reactions and charge transfer accelerates, producing a stable and reversible conductance change consistent with the typical behavior of SnO_2_-based sensors.

In this regard, the responses evaluated at 240 °C and 280 °C were excluded from Figure 7b because at these temperatures, the sensor response exhibits an inversion of the expected reducing-gas trend and unstable signal behavior, as visible in Figure 7a. These responses are not considered reliable and were, therefore, omitted to provide a more representative comparison across the effective operating temperatures.

To the best of the authors’ knowledge, no reliable models or scientific publications exist in the current literature that describe aliphatic VOCs (such as isobutylene) as exhibiting oxidizing behavior (i.e., acting as electron acceptors) on SnO_2_ or other metal oxides at low temperatures.

At low temperatures, aliphatic VOCs such as isobutylene can undergo weak adsorption onto the SnO_2_ surface, primarily through physisorption mechanisms. However, this direct adsorption does not result in significant charge transfer between the gas molecules and the sensing material and, therefore, does not produce a measurable change in conductivity. It is worth noting that the slight increase in resistance observed at low temperatures upon exposure to isobutylene could be tentatively attributed to a weak competitive adsorption effect between the VOC molecules and atmospheric oxygen. Although the reactivity of isobutylene to adsorbed oxygen species is negligible under these conditions, the physisorption of VOCs may marginally influence the equilibrium coverage of oxygen ions, thereby affecting the resistance value. In any case, it is necessary to exceed a temperature of 300 °C to obtain a response to isobutylene fast enough to be exploited for measurement in the application of interest. Below this threshold, the sensor shows very slow behavior. For this gas, there is an optimum temperature around 360 °C; where a very large response is observed, the large response to the tested mixture with a concentration of 0.25 ppm allows for the estimation of a limit of detection (LOD) in the range of tens of ppb.

As expected, for reaction (6), the response time also depends on the concentration of the target gas, whereas the recovery time is independent.

**Figure 9:**
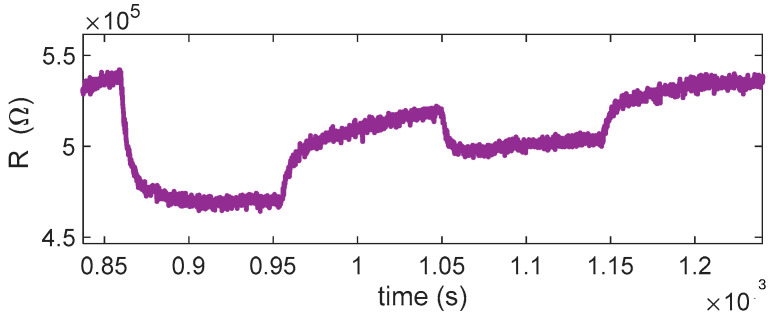
Measured resistance for sensor 7 in the presence of 50% RH as a function of time with target gas concentration pulses followed by recovery phases, 0.5 ppm, 0.25 ppm at 360 °C.

As observed also for NO_2_, the presence of humidity affects the magnitude of the sensor response, which drops by about an order of magnitude (see Figure 8a and Figure 9). Nevertheless, the estimated limit of detection remains about 0.1 ppm, making this sensor suitable for the application.

#### 2.2.3. SO_2_ Gas Sensor Response

Upon exposure to SO_2_, the typical reactions occurring on the SnO_2_ surface can be described as follows [17,18]:

(9) $$S{O_{2}}_{gas}+O_{ads}^{-}\Leftrightarrow S{O_{3}}_{gas}+e^{-}$$

(10) $$S{O_{2}}_{gas}+O_{ads}^{2-}\Leftrightarrow S{O_{3}}_{gas}+{2e}^{-}$$

In this mechanism, SO_2_ acts as a reducing gas: it reacts with chemisorbed oxygen ions, releasing electrons back to the conduction band of SnO_2_, decreasing $\left[Y_{ads}^{-}\right]$ as per Equation (3), and, thus, lowering the sensor resistance. The process is thermally activated, with appreciable sensitivity generally observed above 350 °C, where the catalytic oxidation of SO_2_ to SO_3_ occurs and the reaction rate and recovery remain reasonably fast as to be used for detection applications [19].

**Figure 10:**
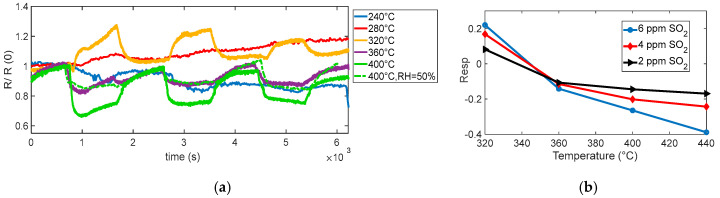
(**a**) Normalized measured resistance as a function of time for sensor 7 during measurement sessions at different operating temperatures (as indicated in the legend) in dry and humid air (target gas concentration pulses followed by recovery phases, 6 ppm, 4 ppm, 2 ppm). (**b**) Measured response (sensor 7) as a function of the operating temperature for the 3 different concentrations, as indicated in the legend.

**Figure 11:**
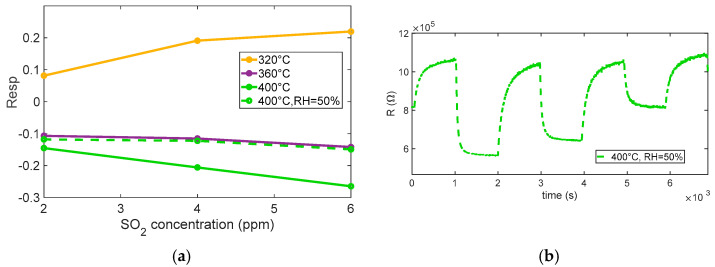
(**a**) Measured response (sensor 7) as a function of SO_2_ concentration for different temperatures and in presence of 50% RH at 400 °C. (**b**) Measured response (sensor 7) as a function of time in humidity environment.

The behavior observed in the performed tests (see Figure 10 and Figure 11) shows a reducing-type response at the highest temperature considered, with an appreciable response becoming evident for temperatures above ~360 °C, as seen in Figure 10. At lower temperatures (below ~320 °C), no considerable response is observed; interestingly, at 320 °C, the response even shows an apparent inversion: the sensor initially behaves as if SO_2_ were oxidizing, increasing the resistance. This inversion effect, also reported for other MOX sensors, can be, as in the case of isobutylene, attributed to competing surface processes and also to the formation of strongly adsorbed species that trap conduction electrons instead of releasing them. At higher temperature, these species tend to desorb or further react, restoring the typical reducing behavior of SO_2_.

In particular, the dynamic behavior visible at 360 °C and especially at 400 °C confirms the enhanced sensitivity of the sensor under these conditions, where the kinetics of surface reactions are accelerated [17].

The test performed at 400 °C under 50% relative humidity shows that humidity slightly affects the response profile, the response appears to be somewhat modified, the baseline resistance value is greatly reduced, and the response appears to be slightly slowed down by the effect of humidity, but no appreciable reduction in SO_2_ sensitivity is observed.

These observations suggest that the optimal operating temperature for achieving a clear and stable reducing response to SO_2_ lies around 400 °C, where surface processes are sufficiently activated to ensure a rapid and reversible sensor response, although care must be taken to account for possible interference from ambient humidity. Notably, at this temperature, the sensor exhibits adequate sensitivity to detect SO_2_ concentrations close to typical outdoor exposure limits (around 4 ppm), making the response time and recovery behavior suitable for practical environmental monitoring applications.

#### 2.2.4. CO Gas Sensor Response

Carbon monoxide is a typical reducing gas whose surface reactions with n-type metal oxide semiconductors, such as SnO_2_, are expected to reduce the film total resistance. The sensing process occurs via electron exchange between the gas molecules and the surface of the oxide, typically in a temperature range starting from 200 °C up to even 430 °C [20]. As mentioned before, in air, molecular oxygen is adsorbed onto the SnO_2_ surface and ionized by capturing electrons from the conduction band according to reaction (7) [21,22].

Upon CO exposure, the expected film behavior involves a combination of reversible chemisorption and irreversible oxidation on the surface, depending on the type of adsorbed oxygen species and operating temperature. We can expect a direct chemisorption of CO [21] according to the following equation:

(11) $$CO_{gas}\Leftrightarrow CO_{ads}^{+}+e^{-}$$

This is equivalent, in charge-transfer terms, to the adsorption of CO followed by electron release to the semiconductor surface [14,22], representing the direct adsorption and oxidation process of CO on SnO_2_ surface sites, S_CO_, involving interaction with surface oxygen vacancies or weakly bonded lattice oxygen. Although not the dominant mechanism, this pathway has been hypothesized and discussed in the literature [23,24] and supported by previous modeling and experimental works of the authors in [25,26]. Conversely, the reaction in the following Equation (12) represents the canonical oxygen-mediated route [21], widely accepted as the principal mechanism governing CO sensing on SnO_2_ surfaces at operating temperatures above ~300 °C, which can be written as

(12) $$CO_{gas}+O_{ads}^{-}\Leftrightarrow C{O_{2}}_{gas}+e^{-}$$

This reaction involves a more stable, irreversible oxidation of CO with surface-adsorbed oxygen species (e.g., O^−^), forming CO_2_ and releasing an electron. Both mechanisms contribute to a decrease in the negative charge trapped at the surface, resulting in a decrease in electrical resistance. In detail, according to reactions (11) and (12), the surface charge density is described by $N_{s}=N_{i}+\left[Y_{ads}^{-}\right]-\left[X_{ads}^{+}\right]=N_{i}+\left[O_{ads}^{x-}\right]-[CO_{ads}^{+}]$; therefore, reaction (11) describes the formation of positive surface charge and reaction (12) the consumption of negative ones. The operating temperatures in this work (≤400 °C) are consistent with the activation range of these surface reactions. From measurements performed in the absence of oxygen, i.e., using pure nitrogen as the carrier gas, we observed a very low response to CO, which indicates that the prevailing reaction is (12).

**Figure 12:**
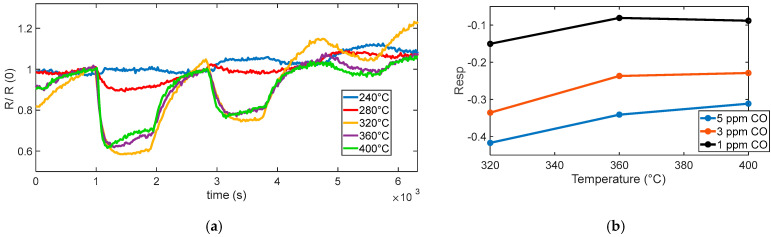
(**a**) Normalized measured resistance as a function of time for sensor 7 during measurement sessions at different operating temperatures (as indicated in the legend) in dry and humid air (target gas concentration pulses of 5 ppm, 3 ppm, 1 ppm of CO followed by recovery phases). (**b**) Measured response for sensor 7 as a function of the operating temperature for the 3 different concentrations, as indicated in the legend.

Figure 12a shows the measured sensor response to various CO concentrations (5 ppm, 3 ppm, and 1 ppm) at different operating temperatures. At low temperatures (below 300 °C in our tests), the sensor exhibited a slow or negligible response, which indicates that the oxygen species are weakly bound and predominantly present in molecular form. A noticeable and consistent response begins to emerge at 320 °C, which corresponds to the activation of chemisorbed oxygen species, primarily O^−^. This temperature range supports fast reactions and ensures high sensitivity, although a significant response drift is observed. It is worth noting that all surface oxidation reactions require activation energy. The discussion in this section refers not to a barrier-free process but rather to the predominance of O_2_^−^ ions as the active oxygen species at low temperatures. According to previous studies [25,26,27], O_2_^−^ species dominate below ~350 °C, while O^−^ ions become prevalent at higher temperatures, which explains the observed temperature-dependent changes in the CO response.

As the temperature increases, the sensor response becomes more stable, and the drift observed at 320 °C is progressively reduced.

Above 360 °C, no substantial further increase in sensitivity is observed; instead, the response tends to plateau and remain stable, suggesting an optimal operating temperature around 400 °C, as summarized in Figure 12b.

**Figure 13:**
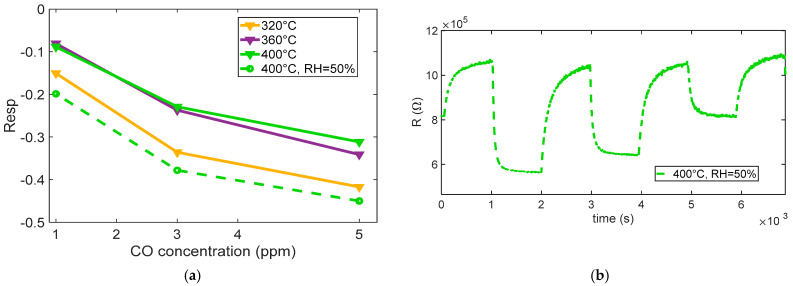
(**a**) Measured response (sensor 7) as a function of CO concentration for different temperatures and in presence of 50% RH at 400 °C. (**b**) Measured response (sensor 7) as a function of time in humidity environment, as indicated in the legend.

Figure 13 summarizes the measured response to the different CO concentrations. The results indicate that, in the presence of humidity, water vapor can significantly influence the CO sensing mechanism on SnO_2_-based MOX sensors. Water molecules adsorb onto the sensor surface and may dissociate to form hydroxyl groups (–OH). These hydroxyl species can either serve as additional reactive sites or modify the surface electronic structure, thereby enhancing the interaction between CO and the sensing layer.

One proposed reaction pathway involves CO reacting with surface hydroxyls as follows [28]:

(13) $$CO_{gas}+2OH_{ads}^{-}\Leftrightarrow C{O_{2}}_{gas}+H_{2}O+e^{-}$$

This reaction contributes to the release of electrons into the conduction band, resulting in a larger decrease in resistance and, consequently, a stronger sensing signal. An additional explanation is that the presence of water vapor can enhance the surface mobility of adsorbed species and improve the kinetics of CO oxidation, thereby increasing both sensitivity and response speed [28].

The enhancing effect of adsorbed water is clearly observed by comparing the curves in Figure 12a with the results in Figure 13b, which show the sensor’s transient response to CO under humid conditions at 400 °C. The response is notably faster than in dry environments and lower-temperature scenarios, with an enhanced gas response that confirms the positive influence of humidity on sensing dynamics.

For CO, the response times appear to be relatively independent of the gas concentration and shorter than the recovery times across all tested levels, indicating that desorption or re-equilibration processes are slower and may represent the rate-limiting step in sensor operation.

## 3. Conclusions and Discussion

This study highlights the complexity of the sensing mechanisms of MOX sensors such as the BME688. The influence of external factors such as temperature and humidity is highly gas-dependent: conditions that enhance the detection of one species may simultaneously reduce the sensitivity or stability to others.

In terms of metrological characterization, the results show that despite large variability in baseline resistance, the normalized responses are consistent across devices, supporting cross-sensor comparability. Each tested gas exhibits an optimum operating temperature in the 360–400 °C range, where sensitivity is maximized and response/recovery times are reduced to a few minutes, making the sensors compatible with mobile and distributed sensing applications. To summarize, for NO_2_, at low operating temperatures (≤ 320 °C), both the response and recovery times exceeded 15 min, indicating slow surface kinetics. Increasing the temperature to 400 °C significantly improved the dynamics, yielding response times of about 8 min and recovery times of 4–10 min, depending on the concentration. Under humid conditions (50% RH), the response times further decreased to 2–4 min, with recovery in approximately 3 min. For isobutylene, response times decreased markedly with temperature—from >15 min below 280 °C to about 1 min at 360–400 °C and 0.5–2 min under 50% RH. Recovery typically required 5–10 min, depending on the gas concentration. For SO_2_, an appreciable response was observed only above 360 °C, with response times of 2–3 min and recovery within 8–13 min at 400 °C. Under humid conditions, the response slightly slowed but remained within a few minutes, while recovery extended up to ~10 min. For CO, the response became appreciable from 320 °C, reaching about 3 min at 400 °C, with recovery typically between 7 and 12 min. In the presence of 50% RH, response times further improved (down to 1.5–2.5 min), with recovery in around 8 min. Overall, these results confirm that the BME688 exhibits temperature-dependent response dynamics, with faster and more reversible behavior at higher temperatures and in humid conditions.

For all gases, the LOD was estimated using the 3σ/slope criterion, where σ is the standard deviation of the baseline signal and the slope is the sensitivity derived from the linear fit of the calibration data. At these optimum temperatures, the LODs obtained for NO_2_, CO, SO_2_, and isobutylene are fully suitable for the intended application, demonstrating that the BME688 can effectively detect pollutant concentrations relevant for air quality monitoring. In fact, in detail, for NO_2_ (≈360–380 °C), the LOD is about 0.1 ppm; for CO (≈400 °C, 50% RH), the LOD is about 0.2 ppm; for SO_2_ (≈400 °C), the LOD is in the range of 0.7–0.8 ppm; and for isobutylene (≈360 °C), the LOD in the low-concentration regime is better than 0.05 ppm (≤50 ppb). Correspondingly, at the respective optimum operating points, the best-case sensitivities are ≈12–18%/ppm (NO_2_, ~360–380 °C, dry), ≈6–9%/ppm (CO, ~400 °C, 50% RH), ≈2–3%/ppm (SO_2_, ~400 °C, dry), and ≈15–25%/ppm (isobutylene, ~360 °C, dry), computed from the low-concentration linear regime after baseline normalization. Regarding stability and repeatability, all experiments were performed using eight identical sensors integrated on the Bosch BME688 Development Kit. Repeated tests under identical conditions (three repetitions) produced highly consistent normalized responses with variations within ±30% across sensors, confirming good reproducibility. Moreover, for each individual sensor, the baseline resistance remained stable over successive exposure/recovery cycles, indicating satisfactory short-term stability under the tested conditions. However, when comparing different sensors on the same board, the normalization of the data is required, based on the knowledge of each sensor’s baseline resistance under reference conditions. This implies that a calibration step is necessary for each sensor to ensure comparability across devices.

Repeated measurements under identical conditions yielded consistent normalized responses with standard deviations of about 20% of the mean value. Such variability is typical of commercial SnO_2_-based devices, reflecting the intrinsic difficulty of reproducing identical surface states due to humidity and adsorption effects. Actually, achieving high-accuracy measurements requires individual sensor calibration and highly specific experimental protocols (e.g., long-term regeneration in dry, pure air at high temperature), conditions with limited practical relevance. In realistic operations, these devices should be regarded as detectors rather than precision instruments, providing reliable trends in pollutant concentration rather than absolute values. The present study contributes to improving their interpretability and practical use, enabling future optimization of selectivity and cross-sensitivity compensation.

All measurements were performed under controlled humidity conditions (50% RH) to reproduce realistic ambient environments while ensuring test reproducibility. The preliminary results indicate that humidity has a significant influence on both response amplitude and recovery speed, but its effects vary depending on the analyte. Specifically, water vapor enhances the dynamics and sensitivity of CO detection through hydroxyl-mediated pathways, while it generally reduces the sensitivity and stability for NO_2_, SO_2_, and isobutylene. This indicates that water interacts with different gases through distinct mechanisms, which must be carefully analyzed and, ideally, modeled for each analyte. In this perspective, a multi-pathway description of adsorption and reaction processes (DIRSI-type models) will be required to account for the complex role of water vapor and to support the development of accurate compensation strategies. Further studies are, therefore, planned for future works to systematically investigate the effect of humidity level on the sensor response.

As a result, while a single sensor can enable the useful specific detection of the presence of pollutants in air, the reliable identification and quantification of specific gases cannot be achieved with a single operating point. Instead, multiple sensors operated at different temperatures are required, exploiting the fact that MOX sensors display reduced or selective sensitivity to certain pollutants under specific thermal regimes.

Temperature profiling strategies, where a sensor is cycled across different operating points, offer a promising path toward improved selectivity. However, defining optimized profiles for complex gas matrices requires an extensive amount of experimental data, demanding substantial time and financial resources. An alternative approach is to leverage physicochemical modeling to accelerate sensor development through simulation. This strategy, though highly challenging, requires modeling not only the fundamental sensing mechanisms but also the kinetics of surface reactions and, critically, the role of water vapor and its interactions with other gases—an aspect widely recognized in the literature as particularly complex.

The authors intend to pursue this modeling-based approach as a way to explore the feasibility of identifying optimized temperature profiles for the selective detection of target pollutants, with the aim of reducing development time and guiding future experimental campaigns. Although this study focused on representative gases relevant to air quality monitoring (CO, NO_2_, SO_2_, and isobutylene), future work will include testing amine-based VOCs such as ethanolamine, triethylamine, and aniline to further assess the selectivity of the BME688 sensor and expand its applicability to broader environmental and biomedical sensing scenarios. The presented results demonstrate that cross-sensitivity among major urban pollutants is significant but manageable through temperature-based operation strategies. More importantly, the BME688 shows adequate sensitivity to detect the concentration ranges relevant to air-quality and exposure monitoring. This confirms its suitability as a compact low-power environmental detector for distributed IoT networks, capable of signaling the presence of pollutants even when selective quantification is not feasible.

## Data Availability

The original contributions presented in this study are included in the article.

## Abbreviations

The following abbreviations are used in this manuscript:

MOX	Metal oxide
LOD	Limit of detection
IoT	Internet of Things
